# Diverse Roles of Annexin A6 in Triple-Negative Breast Cancer Diagnosis, Prognosis and EGFR-Targeted Therapies

**DOI:** 10.3390/cells9081855

**Published:** 2020-08-07

**Authors:** Olga Y. Korolkova, Sarrah E. Widatalla, Stephen D. Williams, Diva S. Whalen, Heather K. Beasley, Josiah Ochieng, Thomas Grewal, Amos M. Sakwe

**Affiliations:** 1 Department of Biochemistry and Cancer Biology, School of Graduate Studies and Research, Meharry Medical College, Nashville, TN 37208, USA; okorolkova@mmc.edu (O.Y.K.); swidatalla13@email.mmc.edu (S.E.W.); swilliams17@email.mmc.edu (S.D.W.); dwhalen15@email.mmc.edu (D.S.W.); hbeasley17@email.mmc.edu (H.K.B.); jochieng@mmc.edu (J.O.); 2 School of Pharmacy, Faculty of Medicine and Health, University of Sydney, Sydney, NSW 2006, Australia; thomas.grewal@sydney.edu.au

**Keywords:** breast cancer, annexin A6, RasGRF2, EGFR, cholesterol, cell growth, cell motility, acquired resistance, tyrosine kinase inhibitors

## Abstract

The calcium (Ca^2+^)-dependent membrane-binding Annexin A6 (AnxA6), is a multifunctional, predominantly intracellular scaffolding protein, now known to play relevant roles in different cancer types through diverse, often cell-type-specific mechanisms. AnxA6 is differentially expressed in various stages/subtypes of several cancers, and its expression in certain tumor cells is also induced by a variety of pharmacological drugs. Together with the secretion of AnxA6 as a component of extracellular vesicles, this suggests that AnxA6 mediates distinct tumor progression patterns via extracellular and/or intracellular activities. Although it lacks enzymatic activity, some of the AnxA6-mediated functions involving membrane, nucleotide and cholesterol binding as well as the scaffolding of specific proteins or multifactorial protein complexes, suggest its potential utility in the diagnosis, prognosis and therapeutic strategies for various cancers. In breast cancer, the low AnxA6 expression levels in the more aggressive basal-like triple-negative breast cancer (TNBC) subtype correlate with its tumor suppressor activity and the poor overall survival of basal-like TNBC patients. In this review, we highlight the potential tumor suppressor function of AnxA6 in TNBC progression and metastasis, the relevance of AnxA6 in the diagnosis and prognosis of several cancers and discuss the concept of therapy-induced expression of AnxA6 as a novel mechanism for acquired resistance of TNBC to tyrosine kinase inhibitors.

## 1. Introduction

Breast cancer is the most common cancer among women in the USA, with an incidence of 63,410 cases of in situ disease, 268,600 new cases of invasive disease, and 41,760 deaths estimated in 2019 [1]. In addition to classification into intrinsic subtypes such as luminal A, luminal B, HER2-enriched, basal-like, claudin-low and normal-like [2,3], breast cancer and triple-negative breast cancer (TNBC) in particular are known to be molecularly heterogeneous diseases. Basal-like breast cancers which are mostly TNBCs, lack or express low levels of the estrogen receptor (ER), progesterone receptor (PR) and human epidermal growth factor receptor-2 (HER2) [4,5]. Based on gene expression profiling of bulk tumors, TNBC tumors are now known to belong to at least four molecular subtypes. These include the immune active basal-like 1 (BL1/BLIA), the immunosuppressed basal-like 2 (BL2/BLIS), the mesenchymal-like (MES) and the luminal androgen-receptor-expressing (LAR) TNBC subtypes [6,7,8]. These mostly high-grade tumors with poor prognosis are particularly prevalent in younger patients, with frequent relapses and metastases to distant organs [9]. About 60–80% of these cancers express variable levels of the epidermal growth factor receptor (EGFR) [10,11], which for many years was considered to be a major oncogene and a promising therapeutic target in these tumors.

The discovery of EGFR as a major oncogene in TNBC sparked intense research on its therapeutic potential and several tyrosine kinase inhibitors (TKIs) and therapeutic monoclonal antibodies (mAbs) targeting this receptor have been developed. Therapeutic monoclonal antibodies against EGFR such as cetuximab bind to the ligand-binding site in the extracellular domain of the receptor. By competing with the receptor ligands, these drugs provoke receptor internalization and degradation, which is accompanied by cell cycle arrest and cell death [12]. Other studies have shown that cetuximab and perhaps other therapeutic monoclonal antibodies induce apoptosis by stimulating the expression of the cell cycle inhibitor p27Kip1 [13]. On the other hand, TKIs such as lapatinib, erlotinib, gefitinib, as well as the more recent generations of these drugs, block the kinase activity of the receptor by competing with ATP binding to the ATP binding pocket in the cytosolic tyrosine kinase domain of the receptor [14]. Some of these TKIs have been approved for the treatment of TNBC, while others are approved for other cancer types, and inhibit tumor growth by promoting cell cycle arrest and apoptosis [15]. However, the use of these EGFR-targeted therapies in the treatment of TNBC and other cancer types have led to dismal outcomes with rapid disease recurrence and metastasis (reviewed in [16]). Although the mechanisms for the frequently acquired resistance to these drugs are continually being unraveled, the failure of these drugs in the treatment of TNBC remains a major challenge. As the recurrence and subsequent disease progression are sustained by residual therapy-resistant tumor cells, remedial approaches will require a better understanding of the mechanisms underlying the ability of the therapy-resistant tumor cells to grow aggressively and/or to become invasive.

Annexin A6 (AnxA6), the largest member (with eight rather than four core domains) of the annexin family of calcium (Ca^2+^)-dependent membrane-binding proteins, is a multifunctional, predominantly intracellular scaffolding protein. In addition, AnxA6 is frequently detected in extracellular vesicles (EVs, ExoCarta exosome database: www.exocarta.org), suggesting that AnxA6 also functions extracellularly. AnxA6 is known to bind to negatively-charged phospholipids, cholesterol, nucleotides as well as a plethora of proteins in a Ca^2+^-dependent manner, and these properties underlie, at least in part, its diverse cellular functions [17,18]. It is increasingly becoming clear that the AnxA6 expression status varies greatly in breast cancer cells as tumor cells with mesenchymal-like phenotypes express higher levels of the protein compared to those with basal-like morphology [19,20]. Although considered to be constitutively expressed in most cell types, AnxA6 expression is also inducible by treatment of tumor cells with a variety of pharmacological drugs [21], and it is differentially expressed in various stages/subtypes of several cancer types including breast cancer [22]. Additionally, it is increasingly becoming evident that disease and/or therapy-associated changes in the expression status of AnxA6 may be useful in the diagnosis, prognosis, as well as in the prediction of patient response to chemotherapy and certain targeted therapeutic options. Here, we highlight the current developments on the potential tumor suppressor and proinvasive roles of AnxA6 in TNBC and other cancers, and how this may be relevant for TNBC diagnosis and prognosis. Finally, we will discuss the novel concept of therapy-induced upregulation of AnxA6 especially in basal-like TNBC cells with low AnxA6 levels (AnxA6-low TNBC cells) as another mechanism for acquired resistance of this hard-to-treat breast cancer subtype to TKIs.

## 2. Molecular Characteristics and AnxA6-Mediated Functions

Based on ample evidence provided over the years, it is now well established that AnxA6 is not only a Ca^2+^-dependent phospholipid-binding protein, but AnxA6 also binds to cholesterol and nucleotides, and serves as a scaffolding protein for several proteins to regulate and/or establish the dynamic association of multifactorial complexes in specialized membrane domains [22,23,24]. These molecular features appear to be critical in the multiple functions of AnxA6 in various cell types and pathological conditions including breast cancer.

### 2.1. Ca^2+^-Dependent Interaction with Cellular Membranes

Elevated concentrations of extracellular Ca^2+^ or activation of Ca^2+^-mobilizing G protein-coupled receptors (GPCRs) and receptor tyrosine kinases (RTKs) lead to an increase in cytosolic Ca^2+^ levels. This transient elevation of intracellular Ca^2+^ levels promotes the translocation of AnxA6 to the plasma membrane and endosomal membranes predominantly via its core domains, as well as to membrane-associated subcellular structures such as microtubules, the actin cytoskeleton, intermediate filaments, membrane lipid rafts, focal adhesions and cell–cell contacts [22,23,24]. The targeting of AnxA6 to these structures, at least in part, underlies the various membrane-associated functions of AnxA6, including regulatory roles in membrane transport along endo-/exocytic and secretory pathways. The Ca^2+^-dependent recruitment of AnxA6 to the plasma membrane has also been shown to contribute to the inactivation of RTKs such as EGFR in A431 epidermal carcinoma cells, HeLa and head and neck cancer cell lines (Fadu, Detroit), by acting as a scaffold for protein kinase C-α (PKC-α) [25,26].

In addition, the upregulation of AnxA6 in a variety of cell lines, including EGFR-overexpressing A431 cells, results in increased association of AnxA6 with late endosomes [19,21,25], which inhibits both cholesterol and endo-/exocytic vesicle trafficking [27,28]. On the other hand, elevated AnxA6 levels have been shown to stabilize activated EGFR and potentially other activated RTKs on the surface of TNBC cells to sustain Ras/MAP kinase signaling and cell proliferation [29]. While these effects may require Ca^2+^-dependent interaction of AnxA6 with distinct effectors followed by the translocation of the complex to the plasma membrane and/or endosomal compartment, the AnxA6-mediated inactivation or sustained activation of RTKs seems to significantly alter the proliferation and motility of tumor cells in a cell type-specific manner. In addition to the regulation of oncogenic receptor function, membrane-localized AnxA6 has also been shown to modulate store-operated Ca^2+^ entry [23], which is a major component of GPCR and RTK function and consequently affects the critical roles of these receptors in cell proliferation, motility and differentiation. Finally, membrane-translocated AnxA6 may also facilitate membrane repair of tumor cells especially following extracellular insults [30,31,32,33]. Therefore, the Ca^2+^-dependent membrane-binding property of AnxA6 triggers numerous Ca^2+^-modulated cellular processes, providing multiple links to the modulation of cell growth, cell motility, differentiation, apoptosis and, presumably, the resistance of TNBC cells to drugs targeting these receptors.

### 2.2. Cholesterol Binding and Subcellular Localization

The abundance and subcellular distribution of cholesterol, an essential component of biological membranes, is strongly influenced by the expression levels and subcellular localization of AnxA6 [32,34]. The possibility that this constitutes a direct interaction of AnxA6 with cholesterol is supported by a proteome-wide mapping of cholesterol interacting proteins that confirmed that several annexins including AnxA6, directly bind cholesterol [35]. Some studies have revealed that the core domain of most annexins is responsible for the cholesterol-mediated effects [36], while others have shown that W343 in the linker region is important for the interaction between AnxA6 and cholesterol [37]. Most strikingly, other cell-based studies revealed Ca^2+^-insensitive, but cholesterol-dependent interaction of cytosolic AnxA6 with late endosomal membranes [38]. Interestingly, a recent study in Niemann–Pick-type C1 mutant cells identified a feedback loop involving selective lysosomal degradation of AnxA6 in the regulation of AnxA6 and cholesterol levels in this subcellular site [39]. On the other hand, upregulation of AnxA6 levels in A431, Chinese hamster ovary and other cell models triggered cholesterol accumulation in the late endosomal compartment [21,27,28]. Overexpression of AnxA6 in TNBC cells has also been linked to the accumulation of cholesterol, especially in late endosomes [21,27]. Together, these studies suggest that AnxA6 contributes to the cellular distribution of cholesterol via this feedback mechanism and presumably, other yet to be discovered mechanisms.

Overall, the association and/or cellular dynamics of AnxA6 and cholesterol appear to underlie the involvement of AnxA6 in not only cell migration [28], vesicle trafficking, exocytosis and endocytosis [27,40], but also in viral uptake, propagation and release [41,42]. The AnxA6/cholesterol paradigm may also be important in membrane repair, cell survival and protection of cells from extracellular insults [43,44]. This includes protection from a cytotoxic surge in intracellular Ca^2+^ induced by Ca^2+^ ionophores or oncogene addiction, which leads to persistent physiological activation of Ca^2+^ mobilizing receptors such as EGFR in TNBC cells [20].

### 2.3. Nucleotide-Binding Characteristic of Annexins

Annexins and AnxA6 in particular, have been shown to bind to nucleotides including ATP and GTP at least in vitro [45,46,47]. This appears to occur via a nucleotide-binding domain located in the N-terminus of nucleotide-sensitive annexins [46]. While the molecular details still remain sparse, the interaction may occur directly [48] or in the case of AnxA7, via bona fide nucleotide-binding proteins such as guanine nucleotide-binding protein subunit beta-2-like 1 (also known as the receptor for activated C kinase 1 (RACK1)) [49]. Earlier studies on the interaction of AnxA6 with nucleotides suggested the existence of two AnxA6 domains within residues 293-301 and 641-649 that potentially bind the phosphate groups of GTP [48]. However, in a follow-up study, the expression of a W343S AnxA6 mutant led to not only diminished GTP binding and GTP-induced ion channel activity, but also the formation of AnxA6 trimers in the presence of GTP [50]. This notwithstanding and given that nucleotides are required for the activation of protein kinases and small GTPases, it is plausible to suggest that the nucleotide-binding characteristic of AnxA6 contributes to cellular functions related to tumorigenesis, endocytosis, exocytosis, vesicular transport and signal transduction pathways. However, whether the interaction of AnxA6 with GTP or other nucleotides is relevant for TNBC progression remains to be fully elucidated.

### 2.4. Scaffolding Functions of AnxA6

Accumulating evidence suggests that the multifunctional role of AnxA6 in cancer may largely depend on its multiple and diverse scaffolding functions. Over the years, it has been demonstrated that in addition to its Ca^2+^, phospholipid, nucleotide and cholesterol-binding properties, AnxA6 also interacts with a vast and diverse number of proteins or protein complexes which to some extent justify its multifunctional role in TNBC and other cell types. The interaction of AnxA6 with F-actin and the F-actin cross-linking protein alpha-actinin [23,51] suggests a role in the remodeling of the actin cytoskeleton, especially during cell adhesion, spreading and motility.

Several members of the S100 family of Ca^2+^ binding proteins are known to associate with cytoskeletal structures including the actin cytoskeleton, intermediate filaments and microtubules. These interactions enable S100 proteins to influence multiple cellular functions, including cell motility [52,53]. The interaction of AnxA6 with some members of the S100 proteins is not only a critical requirement to facilitate the secretion of these S100 proteins [54], but also links the plasma membrane to the cytoskeleton and/or enhances the formation of signaling complexes on biological membranes [55]. In addition, S100/AnxA6 interaction facilitates the bridging of adjacent intracellular vesicles (via annexin molecules) during membrane fusion events [56]. These multiple cellular activities driven by the interaction of AnxA6 with S100 proteins could be critical for the organization of membrane microdomains including lipid rafts, focal adhesions and cell–cell contacts, which independently contribute to enhanced cell motility. 

The binding of AnxA6 to the microtubule-associated protein Tau modulates the distribution of Tau in pathologic conditions [57]. Besides its well-established role in tubulin polymerization and stabilization of microtubules, Tau protein has also been shown to be enriched in metastatic breast tumors [58] and upregulation of Tau protein is associated with resistance to paclitaxel [59] and potentially other taxane-based chemotherapeutic drugs. Although these noncanonical functions of Tau protein are similar to some of the functions listed for AnxA6, whether the Tau/AnxA6 interaction is critical in TNBC metastasis and resistance to taxane-based therapies requires further investigations. 

The interaction of AnxA6 with glycosaminoglycans such as chondroitin sulfate has also been shown to influence cell motility and invasion [60], while AnxA6 expression and its interaction with influenza A virus protein M2 strongly impaired virus release [42,61]. AnxA6 also interacts with the mu subunits of the clathrin assembly protein complex, which further supports its role in endocytosis and vesicular transport [62]. Although AnxA6 may interact with several Ca^2+^ channels to regulate Ca^2+^ influx via its interaction with L-type Ca^2+^ channels, Na^+^/Ca^2+^ exchangers and store-operated Ca^2+^ entry channels, AnxA6 also influences the release of Ca^2+^ from internal stores via its interaction with sarcoplasmic reticulum Ca^2+^-release (SERCA) channels [63,64,65]. Other studies have shown that AnxA6 interacts with extracellular signal effector proteins such as PKC-α [25,66], the Ras GTPase Activating Protein p120RasGAP [67,68], H-Ras [19], Raf-1 [69]; transcription factors such as the p65 subunit of nuclear factor-κB (NF-κB) [70] as well as members of the TBC family of GTPase activating proteins that regulate Rab GTPases [71]. These interactions suggest the involvement of AnxA6 in cellular signaling mechanisms that ultimately lead to tumor cell growth, motility and differentiation.

**Figure 1:**
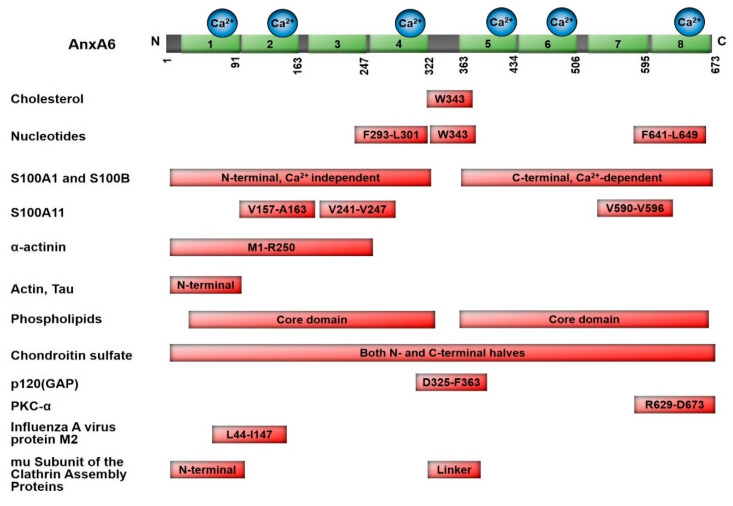
Putative binding domains of some AnxA6 interacting partners. The primary structure of AnxA6 showing the eight annexin repeats and the six potential Ca^2+^ binding sites. The putative binding domains of known interactors including phospholipids, cholesterol, nucleotides and other proteins relative to the primary structure of AnxA6 isoform 1 (NM_001155.5; NP_001146.2; 673 amino acid residues) are indicated (not to scale). See text for further details.

Based on evidence from previous studies, the putative binding domains of cholesterol, nucleotides and some of the known AnxA6-interacting proteins are schematically represented in Figure 1. The annexin core domains have been shown to mediate the interaction of AnxA6 with membrane phospholipids [72] and Tau [57], while the C and N terminal halves of AnxA6 are required for its interaction with S100 proteins [73] and chondroitin sulfate [60]. Actin [74], α-actinin [51] and the mu subunit of clathrin assembly protein [62] have all been shown to interact with the N-terminal of AnxA6. Meanwhile, the minimal interaction segment(s) of some AnxA6 interactors have been mapped. This includes residues 325-363 for p120GAP [75], residues 44 to 147 for influenza A virus M2 protein [61], residues 629–673 for PKC-α [76], residues 157–163, 241–247 and 590–596 for S100A11 [55] and residues 293–301 and 641–647 for nucleotides [48].

Interestingly, a single-residue W343 has been shown to be critical in the binding of both cholesterol [37] and nucleotides [50]. While the list of AnxA6 interacting proteins and/or protein complexes is not yet exhaustive, it is possible that the discovery of novel interacting proteins and the relationship with AnxA6-mediated functions will provide a further premise for its multiple functions in cancer. 

## 3. The Multiple and Diverse Roles of AnxA6 in Tumor Cell Growth and Motility

Over the years, several studies have examined the effects of AnxA6 on hallmarks of cancer such as growth, motility and differentiation in several tumor cell models. However, our understanding of how AnxA6 promotes the progression of TNBC as well as a very diverse set of other cancers [77] remains largely unknown. An emerging concept is that differences in the expression status of intracellular and/or extracellular pools of AnxA6 underlie, at least in part, the distinct phenotypic characteristics of cancer cells and consequently, their propensity to grow rapidly or to become invasive. Here, we review our current knowledge on the role of AnxA6 in cancer progression. 

### 3.1. Altered Expression of AnxA6 in Tumor Cell Proliferation

As a Ca^2+^-dependent membrane-binding protein, AnxA6 has been shown to be involved in several cellular functions that define tumor cell growth. However, this function often appears to be cell- and/or cancer-type specific. In 3T3-L1 preadipocytes, siRNA-mediated AnxA6 knock-down impaired proliferation and differentiation [34]. In vivo proliferation of CD4^+^ T cells, but not CD8^+^ T cells, has also been shown to be impaired in AnxA6^-/-^ relative to WT mice [78]. In human squamous A431 epithelial carcinoma cells, which overexpress EGFR but lack endogenous AnxA6, stable expression of AnxA6 was associated with reduced cell growth [79]. The ectopic AnxA6 expression in these cells led to growth arrest in the G1 phase of the cell cycle, longer doubling times, contact inhibition upon confluence and reduced proliferation when cultured in low serum media [80]. Furthermore, stable AnxA6 expression in A431 xenografts also reduced tumor growth in vivo [79]. Meanwhile, in breast, gastric cancer, hepatocellular and several other cancer types, elevated AnxA6 expression has also been reported to inhibit tumor cell proliferation [22,29,77]. In these tumors and in basal-like TNBCs in particular, AnxA6 appears to act as a tumor suppressor, as reduced expression of AnxA6 led to the rapid growth of xenograft tumors and was associated with poor overall survival of basal-like TNBC patients [20,29]. Interestingly, AnxA6 expression levels are relatively lower in the more aggressive basal-like breast cancer TNBC cell lines than in the more invasive mesenchymal-like TNBC cell lines [20].

The mechanisms underlying the role of AnxA6 in cell proliferation are gradually being unraveled and may include modulation of cell cycle progression [79], regulation of plasma membrane permeability to extracellular Ca^2+^ [23,31,81], inhibition of EGFR and Ras/MAP kinase signaling [19,25,68], interference with cholesterol homeostasis [27,28,71] and protection of cells via facilitation of membrane repair [31,32,33]. Accumulating evidence now suggests that AnxA6 may also affect the growth of tumor cells by modulating glucose and lipid metabolism, with consequences in cellular energy status and consumption. In support of this notion, AnxA6 deficiency compromised alanine-dependent gluconeogenesis during liver regeneration [82]. AnxA6 expression is also associated with fatty-acid-induced lipid droplet formation, with increased lipid droplet numbers and size in a cytoplasmic phospholipase A2α-dependent manner [83]. However, in 3T3-L1 adipocytes, AnxA6 expression decreased cellular triglycerides and adiponectin secretion suggesting that AnxA6 expression in adipose tissues contributes to impaired triglyceride storage and adiponectin release [34]. In TNBC cells, downregulation of AnxA6, which is associated with increased cell proliferation, was accompanied by the upregulation of fatty-acid-binding protein 4 (FAPB4), a protein critical for the import of fatty acids into mitochondria for degradation [20]. Finally, the involvement of AnxA6 in the sensing of intracellular pH during hypoxia [84], mitochondrial morphogenesis [85] and in cellular differentiation [76,86,87], may also contribute to cell viability and growth. While these studies suggest a potential role of AnxA6 in energy metabolism especially in rapidly growing tumor cells, the molecular details and underlying mechanism(s) remain to be fully elucidated.

### 3.2. Tumor Cell Motility and Invasiveness Mediated by AnxA6

Contrary to its role in the proliferation of tumor cells, the expression status of AnxA6 appears to differentially influence the motility of tumor cells in a cell-type- or cancer-type-dependent manner. Overexpression of AnxA6 in human squamous A431 epithelial carcinoma cells is associated with decreased migration in wound healing, as well as reduced invasion in matrigel and organotypic matrices [28]. These anti-invasive properties of ectopically expressed AnxA6 in human squamous A431 epithelial carcinoma cells can be attributed to mislocalization of several SNARE proteins, including SNAP23, syntaxin 4 (Stx4) and Stx6, responsible for the secretion of fibronectin (FN) and the recycling of αVβ3 and α5β1 integrins, respectively [28,40]. The SNARE dysfunction was due to the AnxA6-induced accumulation of cholesterol in late endosomes, leading to obstruction of cholesterol-sensitive exocytic and recycling pathways that deliver FN and integrins to the cell surface [28,40]. These anti-invasive activities of AnxA6 in A431 cells are, however, opposite to the proinvasive effects of AnxA6 in several other cancer cell types including invasive breast cancer cells [22], and migratory neural crest cells [88]. While this reiterates cell-type-specific roles of intracellular AnxA6 pools in cell motility, it is possible that the cell context-dependent requirement of AnxA6 in cell motility is mediated by distinct factors and/or mechanisms. Overexpression of AnxA6 in TNBC cells and presumably other solid tumor types promotes cell motility via activation of the small GTPase Cdc42 [20], which together with other Rho and Rac GTPases are known to promote several different types of cell migration and invasiveness [89]. Other studies have further suggested that the proinvasive roles of AnxA6 in TNBC cells may be linked to the functional status of focal adhesions since the loss of AnxA6 is associated with mislocalization and dysfunction of focal contacts and consequently, loss of invasiveness [22]. Whether this mechanism can be replicated in other cancer cells require further investigations.

Remarkably, several other cell-based studies have suggested a critical role of extracellular AnxA6 in tumor cell motility and invasiveness, as well as cancer metastasis in vivo [20,22,29]. Although a predominantly intracellular protein, AnxA6 is also secreted as a component of extracellular vesicles (EVs, exosomes). These small vesicles are secreted by most cells to facilitate cell–cell communications, and to deliver bioactive molecules that strongly alter the behavior of recipient cells [90,91]. The existence of an extracellular pool of AnxA6 is supported by the detection of AnxA6 in serum exosomes [92] and confirmed in TNBC cell-derived exosomes [90,92]. That the extracellular pool of AnxA6 plays a critical role in cellular adhesion, spreading and motility is also implicated in studies showing that AnxA6 is a cell surface receptor for the abundant liver-derived serum protein Fetuin-A [93], and for chondroitin sulfate [60], both of which important in cell adhesion and motility as components of the extracellular matrix. The proinvasive activity of AnxA6 is further reinforced by the identification of a monoclonal antibody (9E1) against AnxA6 with anti-invasive properties on several aggressive cancer cells including pancreatic, lung squamous and breast cancer cells [94]. These studies presumably suggest that in breast and other cancer types, the proinvasive properties of AnxA6 are mediated by receptor-like properties of AnxA6, as well as by AnxA6-enriched EVs and that the mechanisms may not be limited to the activation of NF-κB and Wnt signaling as recently reported [95].

Despite its Ca^2+^-dependent membrane-binding activity, and the implication of AnxA6 in several membrane-associated events, direct evidence for the involvement of AnxA6 in EV biogenesis and secretion is still lacking. Exosomes are generated from intraluminal membranes (ILVs) in the late endosomal compartment and recent studies identified substantial amounts of AnxA6 to be associated with ILVs [39]. Hence, one could envisage a direct physical association of AnxA6 with the final steps of exosome release at the cell surface, which needs further investigation. However, the notion that tumor cell motility and invasiveness may be mediated by AnxA6-enriched EVs has now been independently demonstrated in breast and pancreatic cancers. These include the findings that AnxA6-enriched EVs from cancer-associated fibroblasts elicited proinvasive properties when taken up by pancreatic and breast cancer cells [96]. Additionally, chemotherapy-stimulated EVs were found to be enriched with AnxA6, and that these EVs facilitated the establishment of breast metastatic lesions in the lungs [97]. While these studies are in strong support for a critical role of AnxA6 in cancer metastasis, this also suggests that AnxA6 enrichment in EVs can be targeted for therapeutic purposes.

### 3.3. Modulation of the Effector Functions of Ca^2+^-Activated Ras Guanine Nucleotide Releasing Factor 2 (RasGRF2) by AnxA6

The demonstration that AnxA6 interacts with PKC-α and p120RasGAP and that these interactions attenuated the activity of EGFR and Ras signaling [19], precludes the role of other components of the Ras signaling pathway such as Ras protein-specific guanine nucleotide exchange factors (RasGEFs). It is possible that other intracellular effectors sensitive to AnxA6 up- or downregulation with roles in cell growth and motility may provide the missing link between AnxA6 expression status and cell growth and motility. Indeed, in a quest to provide a comprehensive picture on the molecular mechanisms underlying the role of AnxA6 in breast cancer cell motility and proliferation, Whalen et al. identified RasGRF2, a Ras protein-specific guanine nucleotide exchange factor (RasGEF), as a major effector of AnxA6-mediated cell growth and motility [20]. In these studies, the expression levels of RasGRF2 and AnxA6 were inversely related in TNBC cells. This is further supported by the relatively high levels of RasGRF2 following downregulation of AnxA6, and the reduced RasGRF2 cellular levels upon AnxA6 overexpression in TNBC cells [20].

As discussed in the preceding sections, although relatively high AnxA6 expression is proinvasive, it has been shown to be antiproliferative in TNBC cells. RasGRF2, on the other hand, promotes cell proliferation via activation of Ras proteins, but inhibits cell motility and invasiveness via inhibition of Rho GTPases, e.g., Cdc42 and Rac1 [20,98,99]. The reciprocal expression of these two proteins is further supported by studies showing that RasGRF2 is activated by a surge in intracellular Ca^2+^ [20,100] and that this is accompanied by its degradation in proteasomes [101]. The activity of RasGRF2 is closely linked to Ca^2+^ homeostasis, in that elevated Ca^2+^ levels stimulate calmodulin-mediated RasGRF2 activation via its IQ domain, with subsequent activation of Ras and/or Rac1 mediated MAP kinase signaling [102]. Overexpression of AnxA6 in TNBC cells not only blocked the EGF/EGFR or Ca^2+^ ionophore stimulated a surge in intracellular Ca^2+^ levels, but also the degradation of RasGRF2 [20,100]. This suggests that the previously demonstrated inhibition of Ca^2+^ influx by AnxA6 [23] underlies the reciprocal expression patterns of AnxA6 and RasGRF2 in TNBC cells. Recent studies from our laboratories revealed that the gene expression profiles of AnxA6-low/RasGRF2-high rapidly growing TNBC cells are distinct from the expression patterns obtained from AnxA6-high/RasGRF2-low invasive TNBC cells [103]. Together, this not only links these proteins to Ca^2+^ mobilization by oncogenic cell surface receptors but also implicates the reciprocal expression of these proteins in TNBC progression and metastasis.

**Figure 2:**
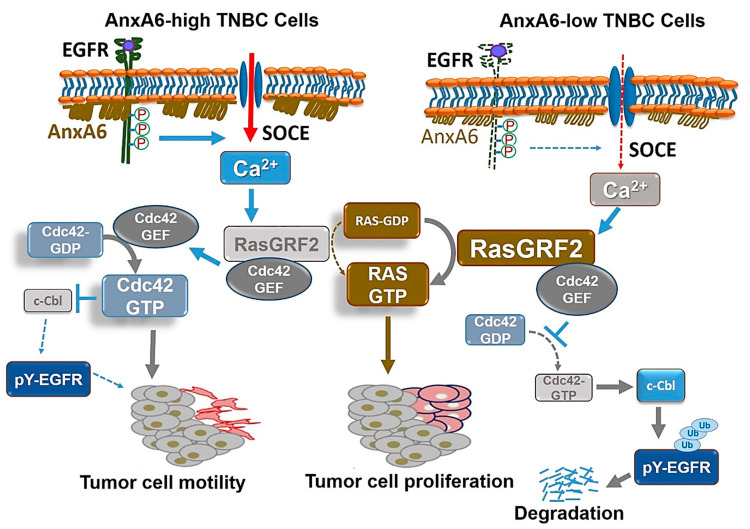
RasGRF2 as an effector of AnxA6-mediated TNBC cell growth and motility. AnxA6 is a predominantly cytosolic protein but upon an increase in intracellular Ca^2+^ levels, it translocates to the plasma membrane where it influences the stability of cell surface receptors, e.g., EGFR and the activity of certain Ca^2+^ channels presumably in membrane lipid rafts. In AnxA6 expressing TNBC cells (left panel), prolonged activation of EGFR leads to a sustained increase in store-operated Ca^2+^ entry (SOCE) and activation, followed by subsequent degradation of RasGRF2. The resulting decrease in cellular RasGRF2 levels enhances the activation of Cdc42 which inhibits the ubiquitin ligase c-Cbl and leads to the stabilization of activated EGFR on the cell surface. The activation of Cdc42 also sustains persistent cell motility. In AnxA6-low TNBC cells (right panel), activation of EGFR is not affected but the activated EGFR (pY-EGFR) is short-lived on the cell surface and therefore, the EGFR signal output is transient. This leads to reduced SOCE, stabilization of RasGRF2 levels and its interaction of Cdc42 GEFs, thereby inhibiting the activity of Cdc42. Reduced activity of Cdc42 enhances the activity of c-Cbl, subsequent ubiquitination of activated EGFR, internalization and degradation by proteasomes. The stabilization of RasGRF2 promotes the activation of the Ras/MAP kinase pathway and enhances cell growth. Abbreviations: AnxA6, annexin A6; EGFR, epidermal growth factor receptor; GEF, guanine nucleotide exchange factor; pY-EGFR, activated EGFR (Tyr-1068); Ras guanine nucleotide releasing factor 2, RasGRF2, SOCE, store-operated Ca^2+^ entry; TNBC, triple-negative breast cancer; Ras/MAP, Ras/mitogen-activated protein kinase.

As shown in the model depicted in Figure 2, modulation of Ca^2+^ influx by AnxA6 in the EGFR/Ca^2+^ influx/RasGRF2 axis represents a critical molecular link between the expression status of AnxA6 in TNBC cells and differences in the invasiveness and proliferation of these cells. In AnxA6-high/RasGRF2-low TNBC cells such as BT-549 cells (Figure 2, left panel), activated EGFR is sustained on the cell surface and the accompanying persistent store-operated Ca^2+^ entry (SOCE) promotes the degradation of RasGRF2. Since RasGRF2 has been shown to interact with RhoGEFs to prevent these GEFs from activating their targets [98], reduced RasGRF2 cellular levels will, therefore, release its inhibition on Rho GTPase GEFs (e.g., Cdc42 GEF), which subsequently activate Cdc42. Cdc42 is also an endogenous inhibitor of the EGFR ubiquitin ligase c-Cbl [104,105], which will lead to inhibition of EGFR ubiquitination, reduced endocytosis and sustained cell surface expression of activated EGFR [29]. Hence, enhanced EGFR activity, together with increased activity of Cdc42 or related Rho GTPases [106] may drive the invasiveness of AnxA6-high/ RasGRF2 low TNBC cells. On the contrary, and as demonstrated by Koumangoye et al., activated EGFR in AnxA6-low/RasGRF2-high TNBC cells, such as MDA-MB-468, is rapidly internalized and degraded [29]. This leads to reduced SOCE, and relatively higher cellular levels of RasGRF2 [20], which not only inhibit the activation of Cdc42 and/or related GTPases, but also activates Ras proteins to drive cell proliferation (Figure 2, right panel). Although this model explains the regulation of the poorly studied receptor tyrosine kinase activated Ca^2+^ influx/RasGRF2 axis by AnxA6 in TNBC cell proliferation and migration/invasiveness, whether this can be exploited for therapeutic purposes remains to be fully investigated.

## 4. Relevance of Annexin A6 in Diagnosis, Prognosis and Therapeutic Interventions

In addition to clinical and pathological characteristics, the most commonly used biomarker panel for intrinsic breast cancer classification is the expression status of ER, PR and HER2. Evaluation of these biomarkers remains the standard method for not only the evaluation of disease prognosis but also current treatment decisions. However, the classification of breast cancer solely on these biomarkers does not accurately represent the complexity of the disease, including very distinct patterns of disease progression, and significant challenges associated with the selection of patients for specific therapies. Serious challenges also remain in identifying relevant therapeutic targets and diagnostic biomarkers for certain breast cancer subtypes including TNBC. Here, we review our current understanding of the potential of AnxA6 as a biomarker for cancer diagnosis, the poor response of TNBC to EGFR-targeted therapies, and the prospects for the detection of AnxA6 as a predictor of the response of TNBC to EGFR-targeted therapies. 

### 4.1. AnxA6 as a Biomarker for Cancer Progression

It has been amply reported that AnxA6 plays a role in the progression of TNBC and other cancer types based on changes in the expression of the protein in various neoplasms (reviewed in [77]). The potential usefulness of AnxA6 as a biomarker for the severity of certain cancer types has also been extensively reported. As indicated in Table 1, this includes studies showing that the upregulation of AnxA6 is an indicator of the progression of ovarian carcinomas [107], women’s thyroid cancer [108], polycystic ovarian syndrome [109], pancreatic cancer [96] and esophageal adenocarcinoma [110]. Other studies have unambiguously demonstrated that AnxA6 may be useful to detect minimal residual disease in B-lineage acute lymphoblastic leukemia [111], the progression of melanomas [112] and squamous cervical cancer carcinogenesis [113]. Meanwhile, some studies have shown that AnxA6 is downregulated in the highly malignant forms of gastric cancer [114], hepatocellular carcinomas [115], cervical cancer [116] and breast cancer [22]. Although these studies emphasize the notion that detection of AnxA6 in several cancers might have diagnostic value, the effects of AxA6 suggest that it may act as either a tumor suppressor or a tumor promoter, depending on the type of cancer and stage of the disease [77,117]. In breast cancer, reduced expression of AnxA6 and its tumor suppressor function is more relevant in TNBC than in non-TNBC subtypes [103], an observation that is consistent with the differences in the malignancy of these breast tumors. In most of these studies, the detection of AnxA6 was carried out by either reverse transcriptase-PCR, immunohistochemistry (IHC) and/or Western blotting. Even though these assays are far from being reliable, it seems feasible that detection of AnxA6 in normal or benign tissues versus malignant tumors may be a reliable indicator of tumor progression and/or malignancy.

### 4.2. Lack of Efficacy of EGFR-Targeted Therapies in the Treatment of TNBC

Although TNBCs lack ER, PR and HER2, 60–80% of these cancers express variable levels of EGFR [5,10,11]. Unlike other cancer types, such as nonsmall cell lung cancer (NSCLC) that express oncogenic EGFR mutants, EGFR in TNBC is rarely mutated, but it is frequently overexpressed (reviewed in [16]). As a potential therapeutic target in TNBC and other cancers, several monoclonal antibodies (mAbs) and TKIs against the EGFR have been developed and tested in breast and other cancer types. These include therapeutic mAbs, such as cetuximab [120,121], and TKIs including lapatinib indicated for metastatic or advanced-stage TNBC [15,122]. Unfortunately, clinical trials to test the effectiveness of these and other EGFR-targeted drugs in TNBC patients have led to modest, poor or incredibly disappointing outcomes with relatively short progression-free survival [120,123].

Yet, EGFR-TKIs have shown promising results in the treatment of other cancers. For example, gefitinib is approved as a first-line treatment for metastatic NSCLC with EGFR exon 19 deletions or the L858R mutant EGFR with or without disease progression [124,125]. While these drugs are often initially effective against cancers with mutated EGFR, some patients acquire resistance due to the development of secondary, often activating mutations in exon 20 (e.g., T790M) [126,127]. Erlotinib has also shown encouraging results in the treatment of pancreatic cancer with EGFR mutations [128,129]. Cetuximab, on the other hand, has been demonstrated to be more effective in the treatment of head and neck squamous cell carcinoma (HNSCC) [130]. Other studies have tested combinations of therapeutic mAbs against EGFR and either TKIs or chemotherapeutic agents including the TBCRC-001 clinical trial of cetuximab and carboplatin [120]. Despite the disappointing results from clinical trials of EGFR-targeted therapies, the possibility that a subset of patients may effectively respond to these drugs remains to be fully explored. However, the challenge is to identify patients who can respond to these drugs with pathological complete response, as well as the need for appropriate biomarkers to monitor disease progression and/or drug efficacy.

### 4.3. AnxA6 as a Predictor of Breast Cancer Recurrence and Response to Therapy

Initial evidence suggesting that AnxA6 levels may influence drug sensitivity and possibly the development of drug resistance is based on the concept that AnxA6 exhibits tumor suppressor activity. In TNBC, high AnxA6 expression is associated with reduced cell growth, while reduced AnxA6 levels promote rapid cell growth. Thus, tumor cells with low AnxA6 expression levels are expected to respond more rapidly to therapeutic interventions, while cells with higher AnxA6 levels may be more refractory to treatment. In support of this concept, Koumangoye et al. demonstrated that reduced AnxA6 expression was associated with poor overall and distant metastasis-free survival of basal-like breast cancer patients and moreover, sensitized TNBC cells to TKIs [29]. This suggests that differential expression of AnxA6 may be useful for the prediction of not only the survival, but also the likelihood of basal-like breast cancer patients to respond to EGFR-targeted therapies. The sensitivity of AnxA6-low TNBC cells to EGFR-TKIs is more likely due to the rapid internalization and degradation of activated EGFR as demonstrated by AnxA6 depletion in TNBC cells [29]. Another recent report revealed that the upregulation of AnxA6 following prolonged treatment of TNBC cells with lapatinib or other EGFR-targeted TKIs was accompanied by accumulation of cholesterol in late endosomes and the development of acquired resistance [21], and this may be predictive of stable and/or progressive disease.

The abundance of AnxA6 in serum and cell-derived EVs also provides an opportunity to predict cancer metastasis in breast and other cancer types. Exosomes originate from internal vesicles of late endosomes/prelysosomes to then fuse with the plasma membrane for subsequent extracellular release. Hence, although the fate of late endosomal AnxA6 and cholesterol in TNBC cells following prolonged treatment with EGFR-TKIs remains to be clarified, it appears plausible to suggest that a proportion of the drug-induced AnxA6 as well as the late endosomal cholesterol may be secreted in EVs. This scenario may support recent studies showing that AnxA6-containing EVs are predictive of metastatic progression in pancreatic cancer [96,97]. Tumor-derived and AnxA6-enriched EVs have also been shown to be prometastatic in mouse mammary tumor virus-polyoma middle tumor-antigen (MMTV-PyMT) and 4T1 mouse models of breast cancer [97]. This suggests that AnxA6 levels in EVs from molecularly distinct breast cancer subtypes and/or chemotherapy-treated patients may be useful to predict the risk of metastasis [96,97] especially in patients who do not achieve a complete pathological response.

### 4.4. Upregulation of AnxA6 Expression and the Development of Acquired Resistance

The mechanisms underlying the poor efficacy of EGFR-targeted therapies are varied and remain poorly understood. As mentioned above, the development of acquired resistance against EGFR-TKIs is common [126,127]. This is often accompanied by overexpression and activation of other receptor tyrosine kinases such as c-MET (also called hepatocyte growth factor receptor), HER2, fibroblast growth factor receptor (FGFR) and AXL and is also associated with resistance to EGFR-TKIs of tumors with EGFR activating mutations (e.g., in NSCLC) [131]. EGFR mAbs are often more effective than TKIs, but resistance to these drugs in various carcinomas is also frequent and is mostly attributed to mutations of effectors downstream of RTKs such as Ras [132], phosphatidylinositol 3-kinase (PI3KCA) and/or loss of phosphatase and tensin homolog (PTEN) [133], as well as activation of BRAF [134]. Since activating mutations of EGFR, Ras and other effectors in the Ras/MAP kinase pathway are rare in TNBC tumors [135], it is possible that the failure of clinical trials and the development of acquired resistance of TNBCs to EGFR-targeted therapies may require distinct and/or unique molecular mechanisms.

Several lines of evidence from our studies and other reports suggest that AnxA6 expression levels are also regulated following the treatment of tumor cells with a variety of pharmacological drugs. This includes studies identifying that exposure of female mice to the suspected endocrine-disrupting xenobiotic plastic precursor bisphenol A, led to AnxA6 upregulation and that this may potentially contribute to the etiology of thyroid cancer in women [108]. Treatment of bone-marrow-derived mesenchymal stem cells with the polyaromatic hydrocarbon fluoranthene also led to AnxA6 upregulation [136]. Furthermore, the hapten challenge of a nonatopic asthma mouse model sensitive to the organofluorine dinitrofluorobenzene also led to increased AnxA6 levels [137]. Although the upregulation of AnxA6 in these studies was detected by proteomic profiling, this nevertheless suggests that detection of AnxA6 together with other coexpressed genes by RT-PCR or IHC, could be useful as novel biomarkers for exposure to these drugs.

We and others have also demonstrated that treatment of breast tumor cells with TKIs [21], nonselective Ca^2+^ channel blockers [103], and the DNA methyltransferase (DNMT) inhibitors 5-aza-2′-deoxycytidine or 5-aza-cytidine [114] led to AnxA6 upregulation. Given that the AnxA6 promoter is heavily methylated, for example in EGFR-overexpressing A431 and ER-negative MDA-MB-468 breast cancer cells, both of which with relatively low AnxA6 levels [19], these effects may be mediated via epigenetic mechanisms including inhibition of DNA methyltransferases and/or histone deacetylases. Within this context, it should also be noted that the human AnxA6 gene is located on chromosome 5q32–q34, with several studies identifying a statistically significant loss of 5q31–q35 in ER-negative tumors and its association with ErbB2 amplification [138,139,140]. It is not clear whether there are ER-negative cell lines with this chromosomal aberration, but it will be interesting to compare the response to therapy of such cell lines to those in which reduced AnxA6 expression could be due to epigenetic mechanisms.

Until recently, it has been unclear whether AnxA6 expression status could be associated with the response of TNBC cells to cytotoxic and/or EGFR-targeted therapies. Strikingly, analysis of AnxA6 expression in stage IV TNBC clinical samples from patients treated with cetuximab and/or carboplatin [120] revealed that treatment of patients with this combination regimen was associated with AnxA6 upregulation. Further analysis showed that the therapy-induced AnxA6 expression was associated with EGFR inhibition rather than chemotherapy in AnxA6-low TNBC cell lines [21]. This finding suggests that unlike treatment with cytotoxic chemotherapeutic drugs such as paclitaxel and carboplatin, treatment of TNBC cells with EGFR-targeted therapies is accompanied by the upregulation of AnxA6, which coincides with an accumulation of late endosomal cholesterol [21]. As indicated above and extensively reviewed elsewhere, cholesterol homeostasis is commonly dysregulated in cancer [141,142] and often accompanied by anticancer drug resistance [143,144,145]. Although AnxA6 strongly influences the intracellular distribution of cholesterol in most cell types [21,146], it is still feasible that AnxA6 expression and high cellular cholesterol levels are independently associated with drug resistance [29]. The latter study suggests that the lapatinib-induced AnxA6 upregulation and concomitant cholesterol accumulation constitutes a novel adaptive mechanism for EGFR-expressing TNBC cells to overcome prolonged treatment with EGFR-targeted TKIs [21]. As further evidence in support of this notion, the withdrawal of lapatinib from lapatinib-resistant cells reversed the expression of AnxA6 to basal levels. Similarly, stable expression of shRNAs targeting AnxA6 in lapatinib-resistant TNBC cells prevented the lapatinib-induced AnxA6 upregulation, suggesting that chronic treatment of TNBC cells with EGFR-TKIs affects AnxA6 mRNA expression rather than the protein stability. Thus far, it remains unclear whether the fate of AnxA6 and cholesterol in the late endosomal compartment of chronic EGFR-TKI treated TNBC cells includes subsequent secretion in EVs. However, these findings provide a strong rationale for further studies to validate the detection of AnxA6 by RT-PCR or IHC along with cholesterol or other markers found in cholesterol-rich and specialized membrane domains (e.g., lipid rafts), as biomarkers for acquired resistance of TNBC to EGFR-targeted therapies and/or other RTK antagonists.

## 5. Conclusions and Future Perspectives

For more than a decade, AnxA6 has drawn considerable interest as a potential suppressor or promoter of cancer initiation and progression in a variety of cancers. This coincides with increasing evidence for AnxA6 being a drug-inducible factor that primarily functions as a scaffolding protein. As high AnxA6 expression levels promote cell motility but attenuate cell growth in TNBCs, the potential for AnxA6 to influence both tumor progression and metastasis has generated great interest. It is also becoming evident that AnxA6 expression levels in various tumor settings most likely reflects the ability of AnxA6 to interact with or indirectly influence the activity of a specific protein or protein complex in a location- and cell type-specific manner; and that this is highly relevant in molecular events that drive cell growth, migration/invasion and differentiation that define carcinogenesis.

The precise role of AnxA6 in drug resistance still remains incomplete. However, the increase in the expression/localization of AnxA6 in late endosomes and the retention of cholesterol in this compartment following prolonged treatment of tumor cells with EGFR-targeting drugs that interfere with Ca^2+^ entry/signaling, provide an exciting first insight implicating high/low AnxA6 levels in drug resistance. Moreover, the upregulation of AnxA6 following chronic treatment of AnxA6-low basal-like TNBC cells with EGFR inhibitors constitutes a novel mechanism for the development of acquired resistance to these and presumably similar drugs targeting other RTKs.

The recent finding that the cellular levels of AnxA6 are inversely related to the levels of RasGRF2 suggests that the reciprocal expression of these proteins in distinct TNBC molecular subtypes underlies, at least in part, the difference in their propensity for growth and/or motility and consequently, tumor malignancy. Together, this provides a rationale for the use of TKIs targeting EGFR/HER2 in combination with inhibition of RasGRF2 to block hyperactive and wild type Ras/MAP kinase pathway-driven triple-negative breast tumors.

Finally, the finding that reduced AnxA6 levels are more relevant in TNBC than in non-TNBC tumors suggests that detection of AnxA6 may not only be useful as a potential biomarker for specific breast cancer subtypes, but also provides promise as a predictor of the response of especially basal-like TNBC to targeted therapeutic interventions. Further studies to exploit these characteristics of AnxA6 are warranted to clearly delineate its usefulness as a diagnostic biomarker and a predictive/prognostic factor in breast and other cancer types.

## Figures and Tables

**Table 1:** Diverse diagnostic, prognostic and therapeutic significance of AnxA6 in cancer progression.

Cancer Type	AnxA6 Expression Status	Diagnostic, Prognostic or Therapeutic Value	Citation
Ovarian carcinoma	Markedly increased in advanced-stage tumors vs. benign controls	Diagnosis of advanced ovarian cancer stages	[107]
Pancreatic cancer	High expression in pancreatic cancer and lung squamous cancer vs. normal tissues	Monoclonal antibody 9E1 as a therapeutic option for invasive cancers	[94]
Pancreatic ductal adenocarcinoma	AnxA6 enriched in EVs from cancer-associated fibroblasts and following chemotherapy	Biomarker and therapeutic target	[96,97]
Esophageal adenocarcinoma	AnxA6 is a component of a 4-protein serum biomarker panel	Noninvasive detection of early tumor stages in patient serum	[110]
Squamous cervical cancer	Expression is increased in cervical intraepithelial neoplasia and microinvasive cervical cancer vs. squamous cervical cancer precursor lesions.	Diagnosis of cervical cancer progression	[113,116]
Acute lymphoblastic leukemia	Highly expressed in B-lineage acute lymphoblastic leukemia vs. normal B-cell progenitors	Diagnosis of B-lineage acute lymphoblastic leukemia	[118]
Breast cancer	Downregulated in EGFR-overexpressing and estrogen receptor (ER)-negative breast cancer cells	Biomarker for EGFR-overexpressing, ER-negative breast cancer	[19]
	Reduced expression in breast cancer tissues, but elevated in invasive breast cancer phenotypes	Biomarker for invasive breast cancer phenotypes	[22]
	Expression status significantly associated with the survival of patients with basal-like breast cancer	Predictive biomarker for basal-like breast cancer patient survival	[29]
	Elevated expression associated with acquired resistance to lapatinib in TNBC.	Predictive biomarker for response to EGFR-targeted therapies	[21]
	Loss of AnxA6 associated with the early onset and rapid growth of xenograft TNBC tumors in mice	Biomaker for TNBC progression	[20]
HER-2/neu-driven mammary tumor	Associated with tumor progression	Biomarker for rapidly growing breast cancer	[119]
Melanoma	Decrease or loss of expression as melanomas progress from benign to malignant phenotypes	Detection of melanoma progression	[112]
Gastric cancer	Downregulated in gastric cancer cells and primary gastric carcinomas	Diagnosis of gastric cancer	[114]
